# Chalcone Isomerase a Key Enzyme for Anthocyanin Biosynthesis in *Ophiorrhiza japonica*

**DOI:** 10.3389/fpls.2019.00865

**Published:** 2019-07-09

**Authors:** Wei Sun, Huan Shen, Hui Xu, Xiaoxin Tang, Ming Tang, Zhigang Ju, Yin Yi

**Affiliations:** ^1^Key Laboratory of State Forestry Administration on Biodiversity Conservation in Karst Mountainous Areas of Southwestern China, School of Life Sciences, Guizhou Normal University, Guiyang, China; ^2^Key Laboratory of Plant Physiology and Development Regulation, School of Life Sciences, Guizhou Normal University, Guiyang, China; ^3^Pharmacy College, Guizhou University of Traditional Chinese Medicine, Guiyang, China

**Keywords:** *Ophiorrhiza japonica*, anthocyanin, chalcone isomerase, transcriptional activity, characterization

## Abstract

Anthocyanins are distributed ubiquitously to terrestrial plants and chalcone isomerase (CHI) catalyzes the stereospecific isomerization of chalcones – a committed step in the anthocyanin biosynthesis pathway. In this study, one gene encoding CHI was isolated from *Ophiorrhiza japonica* and designated as *OjCHI*. Multiple sequence alignments and phylogenetic analysis revealed that OjCHI had the conserved CHI active site residues and was classified into type I CHI group. In order to better understand the mechanisms of anthocyanin synthesis in *O. japonica*, integrative analysis between metabolites and *OjCHI* expression was conducted. The results showed *OjCHI* expression matched the accumulation patterns of anthocyanins not only in different tissues but also during the flower developmental stages, suggesting the potential roles of *OjCHI* in the biosynthesis of anthocyanin. Then biochemical analysis indicated that recombinant OjCHI protein exhibited a typical type I CHI activity which catalyzed the production of naringenin from naringenin chalcone. Moreover, expressing *OjCHI* in Arabidopsis *tt5* mutant restored the anthocyanins and flavonols phenotype of hypocotyl, cotyledon and seed coat, indicating its function as a chalcone isomerase *in vivo*. In summary, our findings reveal the *in vitro* as well as *in vivo* functions of OjCHI and provide a resource to understand the mechanism of anthocyanin biosynthesis in *O. japonica*.

## Introduction

Anthocyanins, a kind of natural pigment, are widespread in plants and predominantly found in flowers, fruits, vegetables, cereals as well as teas (Pervaiz et al., 2017). Among many pigments in nature, anthocyanins assume a critical role because they can confer abundant colors (orange, pink, red, blue, and purple) to different organs of plants such as root, stem, leaf, flower, fruit, and tubers (Grotewold, 2006). Apart from color features, recently, anthocyanins have attracted more interest due to their beneficial effects on human health and plant physiological processes (Kong et al., 2003; Ferreyra et al., 2012). Researches with animals and clinical studies have demonstrated that anthocyanins have an effect in reducing the risk of coronary diseases, stroke and cancer (Thibado et al., 2018). Meanwhile, they also have the biological function for attracting pollinators and the potential to protect plants from getting infected by pathogenic microorganisms (Bradshaw and Schemske, 2003; Konczak and Zhang, 2004). Overall, anthocyanins are interesting secondary plant metabolites as they can be used as plant/food colorants, warning signals, antifeedants, health-promoting agent and so on.

**FIGURE 1:**
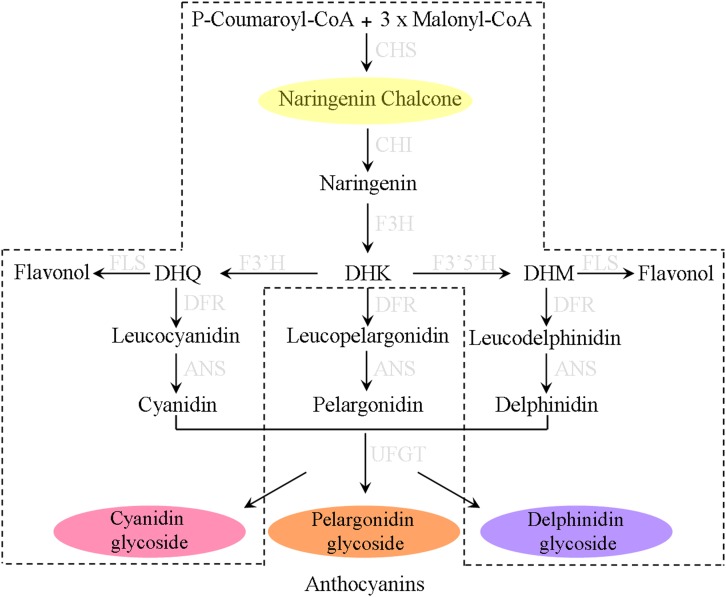
The biosynthesis pathways of anthocyanin pigments in most plants. The proposed pathway in *Ophiorrhiza japonica* is marked with dotted lines.

The biosynthesis of anthocyanins is branched from the phenylpropanoid pathway through the catalysis of various enzymes which are of considerable potential in biotechnological applications (Park et al., 2018). Of these enzymes, chalcone isomerase (CHI, also regarded as chalcone flavonone isomerase) is the second key enzyme in anthocyanin biosynthetic pathway that catalyzes the stereospecific and intramolecular isomerization of naringenin chalcone into its corresponding (2*S*)-flavanones (Figure 1). Although, such an isomerization reaction can conduct spontaneously, the turnover rate is increased 10^7^ fold when CHI participated (Cheng et al., 2018). CHIs in plants can be divided into four types (type I to type IV) depending on their phylogenetic relationships (Ralston et al., 2005). Type I and type II proteins are known as the *bona fide* catalysts with representative CHI enzymatic activity. Type I CHIs are found in most vascular plants and responsible for the formation of general flavonoids (Jez et al., 2000, 2002); while comparing to Type I CHIs, type II CHIs have broader substrate acceptability, besides utilizing naringenin chalcone as substrate, they additionally convert isoliquiritigenin to isoflavonoid which appear to be the specific metabolites in legume (Dixon et al., 1988; Ralston et al., 2005). Unlike type I and type II CHI proteins, both type III and type IV CHIs do not exhibit chalcone cyclization activity and are therefore termed as CHI-like proteins (CHIL). Type III CHIs, widely distributed in land plants and green algae, have been demonstrated to be fatty acid-binding proteins that influence the synthesis and storage of fatty acid in plants (Ngaki et al., 2012). However, the function of type IV CHIs which completely lose the *bona fide* CHI activity remains not well known, though recent studies have showed that this type CHI-fold proteins might serve as the enhancer of flower coloration and flavonoid production in diverse plant species (Morita et al., 2014). In fact, all CHIs have a similar backbone conformation, and type III CHIs are thought to be the common ancestor of *bona fide* CHIs (Jez et al., 2000; Ngaki et al., 2012).

To date, genes encoding CHI have been cloned and identified from many plants, including *D. caryophyllus* (Forkmann and Dangelmayr, 1980), *P. hybrida* (Van et al., 1988), *P. vulgaris* (Blyden et al., 1991), *V. vinifera* (Sparvoli et al., 1994), *P. lobata* (Terai et al., 1996), *M. sativa* (Jez et al., 2000), *L. japonicus* (Shimada et al., 2003), *O. sativa* (Druka et al., 2003), *G. max* (Ralston et al., 2005), *S. medusa* (Li et al., 2006), *G. biloba* (Cheng et al., 2011), *I. batatas* (Guo et al., 2015), *P. lactiflora* (Wu et al., 2017), and *C. nobile* (Wang et al., 2018). Furthermore, their regulatory roles in the biosynthesis of anthocyanins have also been functionally characterized. For example, overexpressing a peony *CHI* gene successfully increased flavonols and flavones content, and reduced the anthocyanin content as well as flower color intensity of transgenic tobacco (Zhou et al., 2014). Similarly, heterologous expression of onion *CHI* in DR-expressing tomatoes generated transgenic fruits with 400- and 260-fold increases levels of anthocyanins in the peel and flesh (Wansang and Jiarui, 2016). These findings reveal the regulatory effects of *CHI* genes on anthocyanins and suggest that it is possible to obtain desirable agronomic traits through manipulating this enzyme.

*Ophiorrhiza japonica* is a perennial herbal plant belonging to Rubiaceae. This herb is a precious Chinese medicinal plant, which can produce flavonoid, camptothecin and other medicinal ingredients. It has a wide distribution in China and is commonly used in the treatment of ulcers, poisonous wounds, leprosy, rheumatism and so on. Recently, anthocyanin biosynthesis and its related genes have been well studied in various plants (Petroni and Tonelli, 2011). However, the accumulation and biosynthesis of anthocyanin in *O. japonica* is still unknown. In the present study, we perform the first comprehensive study on the molecular characterization of *CHI* and anthocyanin analyses in *O. japonica*. Firstly, the chemically characterization of anthocyanins as well as their content in different tissues were measured. Then the *OjCHI* expression and its functional characterization in relation to anthocyanin biosynthesis were well investigated. Overall, we identify one chalcone isomerase-fold protein, which is critical for anthocyanin production. And the results presented in this work not only further our understanding of the molecular mechanism of the anthocyanin biosynthesis in *O. japonica*, but also open up the possibility of synthesizing high-value plant anthocyanins and their derivatives using biochemical and biotechnological methods.

## Materials and Methods

### Plant Materials

*Ophiorrhiza japonica* materials used in this paper were collected on the mountain in Shibing, Guizhou Province. Flowers of *O. japonica* at different developmental stages as well as roots, stems, leaves, scapes, and calyxes were obtained in October, 2017, then all collected tissues were flash frozen by liquid nitrogen and kept at -80°C for later analyses. *CHI* mutants (*tt5*, SALK_034145) obtained from the Arabidopsis Biological Resource Center were in the Columbia ecotype background and grown under the conditions as described before (Sun et al., 2016). For Anthocyanin and RT-PCR analysis, 7-day-old Arabidopsis seedlings cultured on anthocyanin gene induction media (half-strength MS medium supplemented 3% sucrose) were harvested and stored at -80°C.

### Measurement of Anthocyanin and Flavonol Extracted From *O. japonica*

The composition and contents of anthocyanin and flavonol were determined by high-performance liquid chromatography (HPLC). For extraction, 0.1 g flour of each sample was homogenized in 1 ml extraction solvent (H_2_O:MeOH:HCl; v/v/v, 75/24/1) for 12 h in darkness at 4°C. Then the extracts attained were centrifuged at 12,000 rpm for 10 min, and the supernatant was passed through a 0.22 μm reinforced nylon membrane filter before subjecting to HPLC identification. The Shimadzu HPLC system with API 2000 mass spectrometer was used for qualitative analysis according to the protocol as described by Sun et al. (2015). Anthocyanin and flavonol contents were calculated based on the external standard curve calibration of cyanidin 3-*O*-glucoside and quercetin-3-*O*-glucoside standards (Sigma-Aldrich, St. Louis, MO, United States) (Fanali et al., 2011). Each sample used for HPLC measurements was determined with three biological replicates.

### Cloning of *OjCHI* and Phylogenetic Analysis

Based on the transcriptome data of different tissues of *O. japonica* measured before, a total of five sequences of the *CHI* gene from *O. japonica* were identified through blastn alignment with reference genes of proximal species and Arabidopsis CHI protein sequence. Then comparative analysis was conducted by using the following database: national center for biotechnology information (NCBI) and the results indicated that the sequence of unigene (*OjCHI*) (Unigene35425_All) showed the highest similarity to *CHI* genes from other plants. Therefore, the specific primers (OjCHIF1 and OjCHIR1) were designed from sequence information of *OjCHI* gene. To isolate the total RNA, flowers of *O. japonica* at stage 4 were ground into powder and extracted by RNA pure Plant Kit (CWBIO, China). Then the cDNA was synthesized from 1.0 μg total RNA using EasyScript One-Step gDNA Removal and cDNA Synthesis SuperMix (TransGen, China). Subsequently, for obtaining the full-length sequence of *OjCHI*, its complete open reading frame (ORF) generated by RT-PCR was cloned into pMD18-T vector and sequenced by Sangon Biotech (Shanghai, China). All the primers used in this work are listed in Supplementary Table S1.

### Quantitative RT-PCR Analysis

Total RNA isolation and cDNA synthesis were performed as described above. qRT-PCR primers specific for *OjCHI* and *actin* were designed by using IDT^1^ and listed in Supplementary Table S1. Quantitative RT-PCR analyses were carried out on ABI 7500 System using *TransStart*^®^ Green qPCR SuperMix (TRANSGEN, China). Each PCR reaction in 20 μl volume included 10 μl 2× *TransStart*^®^ Green qPCR SuperMix, 0.8 μl forward and reverse primers, 1 μl template cDNA and 9.2 μl Nuclease-free water. To confirm purity of the PCR products, melting curve analysis and sequencing was employed. The 2^-ΔΔCT^ method was used for *OjCHI* expression analysis through normalizing to the *actin* gene from *O. japonica* (Livak and Schmittgen, 2001). Three independent biological replicates were conducted for each experiment sample.

### Expression and Purification of Recombinant *OjCHI*

The open reading frame of *OjCHI* was amplified using the primers (OjCHIF3 and OjCHIR3) in Supplementary Table S1 and subcloned into the pET-32a expression vector. After verification by sequencing, the recombinant construct as well as the empty vector were transformed into *Escherichia coli* strain BL21, respectively. The overnight bacterial cultures obtained from a single transgenic colony were diluted into LB medium and grown to OD_600_ = 0.6, at which point 0.35 mM isopropyl β-d-thiogalactoside was added to induce recombinant protein expression at 30°C for 10 h. Then the cells were harvested through centrifugation at 6,000 rpm for 10 min and disrupted by sonication on ice. The His_6_-tagged recombinant proteins were purified using Ni-NTA pre-packed column (TransGen, China) following the manufacturer’s recommendations and its purity was finally tested by SDS-PAGE.

### Enzymatic Activity Assay

Chalcone isomerase activity for production of naringenin from naringenin chalcone was performed in a total volume of 50 μL containing 50 mM potassium phosphate (pH 7.5), 50 μM substrate and 10 μg purified recombinant OjCHI protein. Soluble protein extract from induced BL21 containing empty pET-32a vector was used as a control. After incubating at 30°C for 5 min, the reaction mixtures were terminated and extracted twice with 100 μL ethyl acetate, and centrifuged at 12,000 rpm for 10 min. Subsequently, the supernatant was subjected to high-performance liquid chromatography analysis using a Shimadzu HPLC system. The mobile phases were composed of 50% methanol, 48% water, and 2% acetic acid at a flow rate of 0.8 ml per minute. The enzymatic products were detected at 304 nm with a column temperature of 40°C.

### Plant Transformation and Metabolite Analysis of Transgenic Seedlings

The cDNA of *OjCHI* was amplified by PCR with primers OjCHIF4 and OjCHIR4 (Supplementary Table S1), and cloned into the binary vector pBI121. The resulting construct containing *OjCHI* was introduced into *A. tumefaciens* strain GV3101 through freeze-thaw method, after that, the standard flower dip protocol was used for Arabidopsis *tt5* mutant transformation (Clough and Bent, 1998). T2 generation seeds and their seedlings grown on anthocyanin gene induction media were selected for phenotypic investigations and metabolite analysis. To confirm *OjCHI* expression, RT-PCR analysis was performed with Arabidopsis *actin-1* gene as internal reference (Penninckx et al., 1996). Qualitative and quantitative analysis of anthocyanins and flavonols in transgenic Arabidopsis was conducted using the methods described above.

## Results

### Characterization of *CHI* Gene From *O. japonica*

**FIGURE 2:**
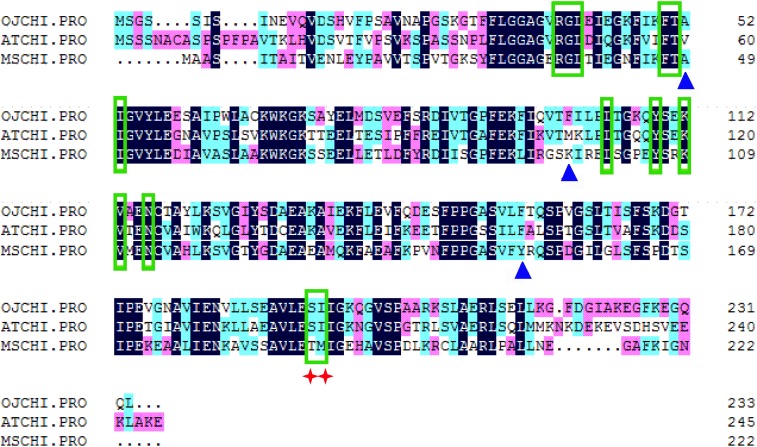
Sequence alignment of OjCHI with AtCHI (*Arabidopsis thaliana*, P41088) and MsCHI (*Medicago sativa*, P28012). Residues for binding (*2S*)-naringenin are shown with green boxes, and those for hydrogen bond network in active site are shown with blue triangles. The red star identifies residues postulated to determine substrate preference for naringenin chalcone and isoliquiritigenin.

**FIGURE 3:**
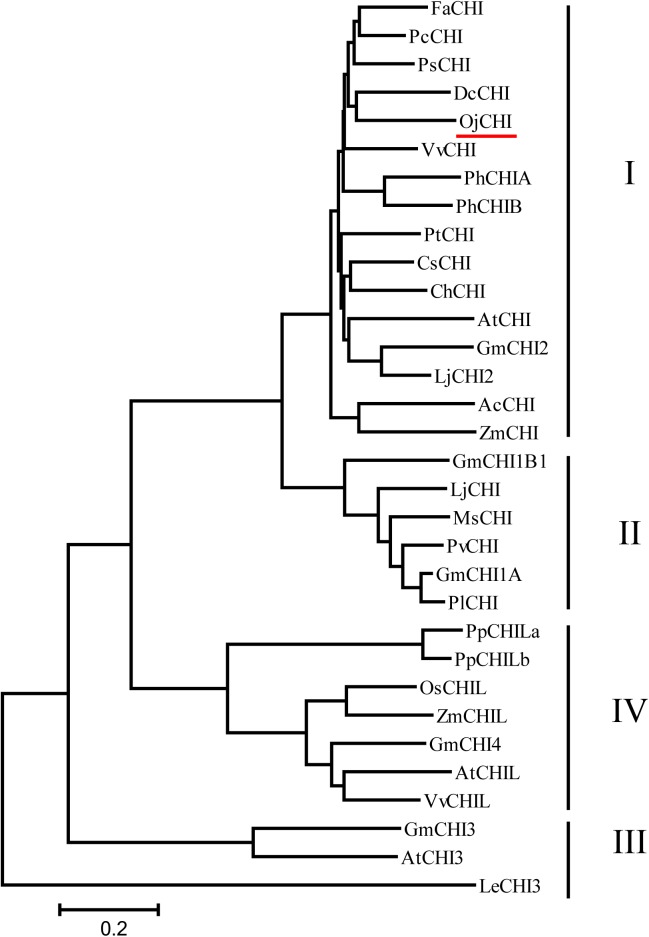
Phylogenetic analyses of the deduced amino acids of OjCHI and CHIs from different plant species. GenBank accession numbers are as follows: AtCHI (*Arabidopsis thaliana*, P41088), MsCHI (*Medicago sativa*, P28012), PsCHI (*Paeonia suffruticosa*, ADK55061), ZmCHI (*Zea mays*, CAA80441), VvCHI (*Vitis vinifera*, P51117), CsCHI (*Citrus sinensis*, BAA36552), GmCHI2 (*Glycine max*, AY595415), LjCHI2 (*Lotus japonicus*, Q8H0G1), GmCHI1B1 (*Glycine max*, AY595414), LjCHI (*Lotus japonicus*, AJ548840), PvCHI (*Phaseolus vulgaris*, P14298), GmCHI1A (*Glycine max*, AY595413), PlCHI (*Pueraria lobata*, Q43056), PpCHILa (*Physcomitrella patens*, XP_001773128), PpCHILb (*Physcomitrella patens*, XP_001769093), VvCHIL (*Vitis vinifera*, XP_002280158), AtCHIL (*Arabidopsis thaliana*, NP_568154), GmCHI4 (*Glycine max*, AY595417), OsCHIL (*Oryza sativa*, NP_001065587), ZmCHIL (*Zea mays*, NP_001151452), GmCHI3 (*Glycine max*, AY595416), PhCHIA (*Petunia hybrida*, AAF60296), PhCHIB (*Petunia hybrida*, CAA32730.1), AcCHI (*Allium cepa*, AY700850), ChCHI (*Gossypium hirsutum*, ABM64798), DcCHI (*Dianthus caryophyllus*, Q43754), AtCHI3 (*Arabidopsis thaliana*, AY084729), FaCHI (*Fragaria ananassa*, Q4AE11), PtCHI (*Populus trichocarpa*, XP_002315258), PcCHI (*Pyrus communis*, A5HBK6), and LeCHI3 (*Lycopersicon esculentum*, AY348871).

The ORF of *CHI* from *O. japonica* was successfully isolated and designated as *OjCHI*, which encodes a polypeptide of 233 amino acids long, with a calculated isoelectric point of 4.95 and a predicted molecular mass of 25.018 kDa. Sequence alignment revealed that the deduced polypeptide sequences of OjCHI was aligned well with the established type I and type II CHIs of Arabidopsis and *Medicago sativa*. The overall identities of OjCHI to Arabidopsis and *Medicago sativa* are 56.91 and 51.07%, respectively, at the amino acid level. Furthermore, OjCHI also shares many conserved residues with AtCHI and MsCHI. For example, the active site residues proved in MsCHI for binding (*2S*)-naringenin are conserved in OjCHI. Importantly, the residues proposed to determine substrate preference in type I CHIs (AtCHI1: Ser211 and Ile212; OjCHI1:Ser193 and Ile194) are also presented in OjCHI (Figure 2). These results suggest that OjCHI is the member of CHI family. Phylogenetic analysis was then conducted based on CHIs from different plant species using the neighbor-joining method. As shown in Figure 3, the established tree consisted of four branches, and OjCHI was classified into type I CHI family, which includes AtCHI/tt5 catalyzing the stereospecific cyclization of naringenin chalcones.

### Anthocyanins Analysis

**FIGURE 4:**
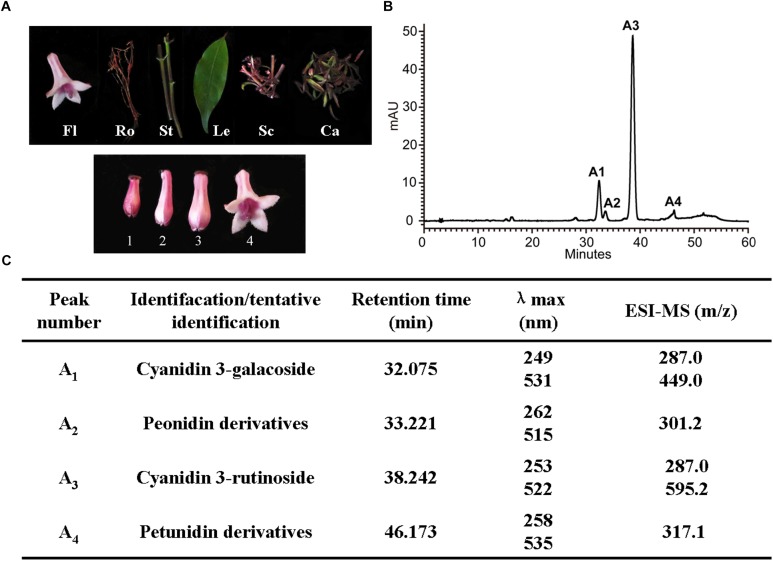
Anthocyanin component analyses in *O. japonica*. **(A)** The phenotypes of different samples. Fl, flowers; Ro, roots; St, stems; Le, leaves; Sc, scapes; Ca, calyxes. One to four, represent the flowers of different developmental stages. **(B)** High performance liquid chromatography (HPLC) profiles of anthocyanins in calyxes. **(C)** The anthocyanin profiles in acidic MeOH-H_2_O extracts of the *O. japonica*.

To understand the dynamic change trends of anthocyanin in *O. japonica*, anthocyanin in different tissues (flowers, roots, stems, leaves, scapes, calyxes) and flowers at different developmental stages were identified and quantified (Figure 4A). Based on HPLC results, a total of four kinds of anthocyanin (A1–A4) were detected in *O. japonica*, and these anthocyanins were then identified as cyanidin 3-galactoside, peonidin derivatives, cyanidin 3-rutinoside, and petunidin derivatives according to the MS analysis (Figure 4B,C). Later, quantitative analysis showed that the contents of cyanidin 3-rutinoside (A3) were the most abundant anthocyanin all the times (accounting for 60.9–100% of the total anthocyanin), and its highest accumulation level was found in calyxes and stage 1 (Supplementary Figure S1). However, among the basic anthocyanins, pelargonidin glycosides were not detected.

### The Relationship Between *OjCHI* Expression and Accumulation of Anthocyanins in *O. japonica*

**FIGURE 5:**
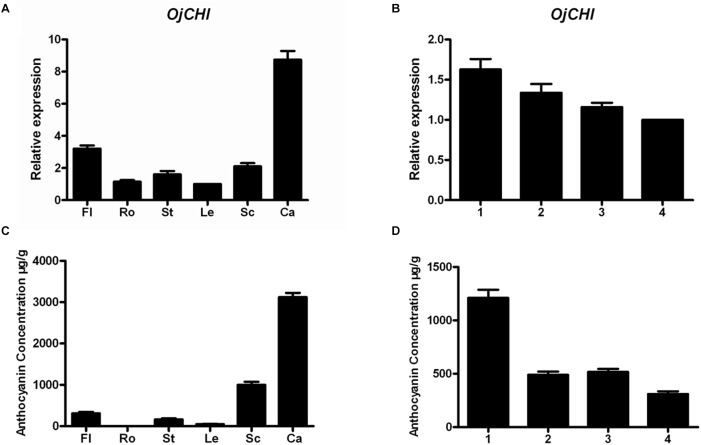
Gene expression of *OjCHI* and anthocyanin accumulation in *O. japonica*. **(A,B)** Relative transcript levels of *OjCHI* in different tissues and flowers at different developmental stages. **(C,D)** Quantitative analyses of total anthocyanins in different tissues and flowers at different developmental stages. Data represent means ± SD of three biological replicates.

The transcription levels of *OjCHI* and the amount of total anthocyanins accumulation were investigated in different tissues and flowers at four developmental stages. Transcripts of *OjCHI* were detected in all tested tissues, but its expression was tissue specific. As shown in Figure 5A, *OjCHI* expressed dramatically higher in calyxes than in other tissues and exhibited almost equal expression in roots and leaves. Furthermore, the mRNA levels of *OjCHI* were flower development-dependent, gradually declined during flower development and showed maximum expressions at stage 1 (Figure 5B). In a similar way, the total contents of anthocyanins displayed more consistency to the *OjCHI* expression not only in different tissues but also during the whole flower developmental stages. As seen in Figure 5C, anthocyanins were also detected abundantly in calyxes, and during the flower development, its levels continued to decrease gradually from stage 1 to a minimum at stage 4 (Figure 5D). Taken together, these results suggest that the expression of *OjCHI* appears to be one of the key factors determining anthocyanin accumulation pattern in *O. japonica*.

### Functional Analysis of Recombinant *OjCHI in vitro*

**FIGURE 6:**
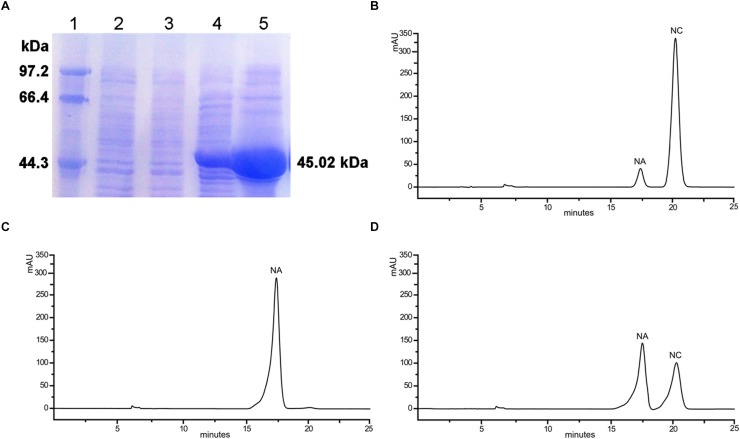
*In vitro* enzymatic assays of the recombinant OjCHI. The assays were conducted with naringenin chalcone as substrate. **(A)** Expression of OjCHI in *E. coli*. (1) Maker (2) Total soluble protein from *E. coli* expressing pET-32a (+) vector (3) Total soluble protein from *E. coli* expressing OjCHI prior to induction with IPTG (4) 10 h after induction (5) Purified OjCHI. **(B)** Naringenin chalcone standard. **(C)** HPLC profiles of the reaction products of OjCHI. **(D)** The control (empty pET-32a vector).

To examine if OjCHI has isomerase activity under *in vitro* conditions, its coding sequence was cloned into pET-32a, an expression vector with a His-tag, yielding pET32a-OjCHI. Based on induction by IPTG at low temperature, the recombinant OjCHI was expressed as a major soluble protein. Subsequently, OjCHI protein was purified by Nickel-NTA agarose and the size was in agreement with predication (Figure 6A). OjCHI catalytic activities was then assayed with naringenin chalcone as substrate, 5 min later, all the naringenin chalcone was catalyzed to a product with a similar retention time to naringenin (5-hydroxychalcone) as observed by HPLC (Figure 6B,C). In contrast, the control reaction, in which the protein from *E. coli* carrying the pET-32a vector was also incubated with naringenin chalcone, but showed residual substrate and less quantity of spontaneous product, implying a low level non-enzymatic conversion to naringenin (Figure 6D). Thus, these experimental data indicate that OjCHI is capable of metabolizing naringenin chalcone to naringenin and shows a typical type I CHI-cyclization activity.

### Complementation of the *tt5* Mutant With *OjCHI*

**FIGURE 7:**
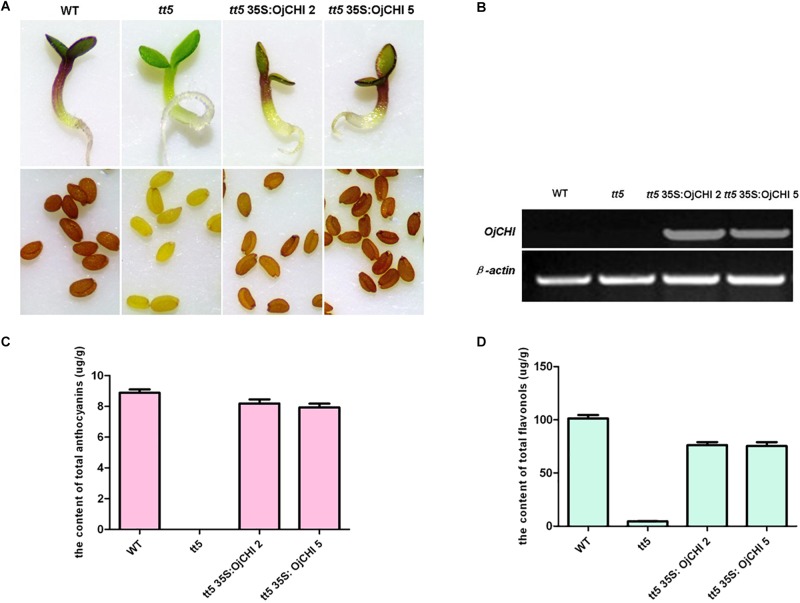
Complementation of *CHI* function in Arabidopsis *tt5* mutant. **(A)** Phenotype of wild type (WT), *tt5* mutant and *tt5* 35S: OjCHI lines in their seedlings and testas. **(B)** Expressional analyses of the *OjCHI* gene by RT-PCR in WT, *tt5* mutant and *tt5* 35S: OjCHI lines. **(C,D)** Contents of anthocyanins and flavonols in Arabidopsis seedlings. Data correspond to means of three biological replicates.

To validate the *OjCHI* function during flavonoid biosynthesis, the *OjCHI* gene was overexpressed in Arabidopsis *tt5* mutant under the control of CaMV 35S promoter. The *tt5* mutant failed to accumulate condensed tannins which confer a yellow color on their testas, and the synthesis of anthocyanin pigments in *tt5* was also blocked in their cotyledon and hypocotyls caused by the mutation in CHI. Altogether, ten independent kanamycin-resistant transgenic lines were obtained. As in the wild type, seeds from T2 transgenic plants expressing *OjCHI* were brown, and the cotyledons as well as hypocotyls of the seedlings showed restoration of purple coloration (Figure 7A). Furthermore, to confirm the overexpression of *OjCHI*, RT-PCR was performed, and the results revealed that the *OjCHI* gene was successfully expressed (Figure 7B). Quantification of anthocyanin and flavonol indicated that anthocyanin and flavonol levels of transgenic seedlings were significantly higher than those of *tt5*, which accounted for 89.3–92.1% and 73.9–75.2% of the total anthocyanin and flavonol content in wild type, respectively (Figure 7C,D).

**FIGURE 8:**
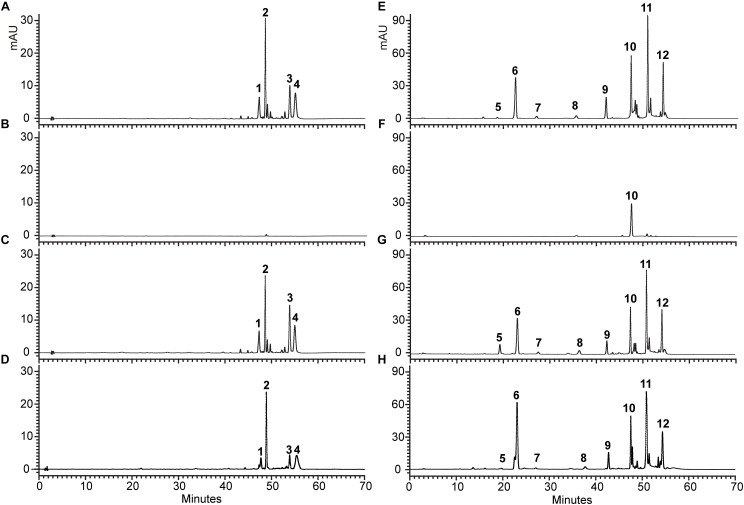
HPLC profiles of anthocyanins and flavonols in Arabidopsis seedlings. **(A–D)** Absorbance at 520 nm for analysis of anthocyanins. **(E–H)** Absorbance at 360 nm for analysis of flavonols. **(A,E)** HPLC chromatograms of the samples from seedlings of WT. **(B,F)** HPLC chromatograms of the samples from seedlings of *tt5* mutant. **(C,G)** HPLC chromatograms of the samples from seedlings of *tt5* 35S: OjCHI 2. **(D,H)** HPLC chromatograms of the samples from seedlings of *tt5* 35S: OjCHI 5.

Additionally, for examining the change of anthocyanin and flavonol in transgenic seedlings in more detail, HPLC analysis was conducted, and the structure of anthocyanin and flavonol was further confirmed by LC-MS/MS (Supplementary Table S2). As shown in Figure 8, *tt5* mutant had an untraceable and reduced peak area for the peaks of anthocyanin and flavonol comparing with the wild type control. As expected, transgenic seedlings expressing *OjCHI* displayed restoration of these peaks, though the content of anthocyanin and flavonol was lower than wild type Arabidopsis. Overall, the flavonoid analysis data demonstrate that the *OjCHI* gene could encode a functional CHI, which is fully functional for the biosynthesis of anthocyanin and flavonol *in vivo*.

## Discussion

Anthocyanins, the main products of flavonoid pathway, are ubiquitous in many plants, having a strong impact on their pigmentation, taste and resistance. CHIs are known as the key enzymes which exhibit vital regulatory function during the anthocyanin biosynthesis (Grotewold, 2006; Zhou et al., 2014). Given the importance of this enzyme, comprehensive study of CHI involved in flavonoid biosynthesis has become one of the hot spots in plant secondary metabolism research. In present work, the full-length cDNA of *O. japonica CHI* was successfully cloned, and its amino acid sequence displayed high identity to CHI from other plants, indicating that *OjCHI* is one of the members of CHI family. Structure-function analysis of MsCHI revealed that several critical amino acid residues that important for CHI catalytic action were conserved in all plants (Jez et al., 2000). And the kinds of amino acid residues at position 190 and 191 were proposed to be important in determining substrate preference and classification of CHI. Previous study revealed that amino acid residues at position 190 and 191 of type II CHI were Thr and Met, while type I CHI replaced them with Ser and Ile (Forkmann and Dangelmayr, 1980; Jez et al., 2000). After aligning the amino acid sequences of *OjCHI* with typical type I (AtCHI) and type II (MsCHI) CHI, we found OjCHI had all conserved amino acid residues and contained Ser and Ile at position 190 and 191, respectively (Figure 2). These results strongly suggest that OjCHI might be a type I CHI which is essential for anthocyanin and other flavonoid compounds biosynthesis in plants. Meanwhile, phylogenetic analysis of OjCHI (Figure 3) also further hints its function as type I CHI for anthocyanin synthesis (Winkel-Shirley, 2001).

Consistent with the results from sequences comparison and phylogenetic analysis, biochemical assays of OjCHI showed that nearly all the substrate (naringenin chalcone) was catalyzed into a product with its retention time identical to that of naringenin (Figure 6). This reaction profile is similar to that of GbCHI in *Ginkgo biloba* and confirms OjCHI indeed belong to the type I CHI which is necessary for the production of anthocyanin (Cheng et al., 2011). To best of our knowledge, the *bona fide* CHIs are divided into two groups (type I and type II) on the basis of their substrate specificity and catalytic activity, moreover, the type II CHI proteins are regarded as “legume-specific” CHIs (Ralston et al., 2005). But recently, research of Cheng et al. corroborated that the type II CHI proteins also emerged in liverwort as well as other ancient land plants species, and the type I CHI proteins with Ser and Ile/Met at position 190 and 191 from higher plants were likely evolved from the primitive *bona fide* type II CHIs (Cheng et al., 2018). Therefore, it will be interesting and necessary to perform more detailed analysis about CHIs in *O. japonica* through using more advanced characterization techniques to deepen our understanding on the CHIs evolution and flavonoid metabolism.

Anthocyanin qualitative analysis indicated that four kinds of anthocyanins had been detected in *O. japonica* but lacked pelargonidin derivatives – based on this, the proposed anthocyanin pathway was listed in Figure 1. According to previous reports, this interruption of anthocyanin pathway was ascribed to the substrate specificities of dihydroflavonol 4-reductase (DFR). Actually, studies of DFRs from *Petunia hybrida* and *Angelonia angustifolia* found, due to the DFR substrate specificities, both plants unable to synthesize pelargonidin-based anthocyanins (Mol et al., 1998; Gosch et al., 2014). On the other hand, it is likely that competitive advantage of FLS compared with DFR in dihydrokaempferol (DHK) utilization may be another cause for the deficiency of pelargonidin derivatives (Gu et al., 2018).

Expression levels of *CHIs* have been investigated in various plants, and their transcripts sometimes are congruent with the accumulation of anthocyanins in target tissues (Shoeva et al., 2014). However, the transcript expression studies of *CHI* gene in *O. japonica* have not been reported yet. Therefore, transcript analyses of *OjCHI* in different tissues were conducted. As shown in Figure 5A, *OjCHI* was actively transcribed in all organs examined, and showed relatively low expression in the root, which is similar to that of *CHI* in herbaceous peony (Zhao et al., 2012). Previously, it was reported that expressions of leguminous *CHI* genes were always strong in root, because they would act as signaling molecules to play crucial roles during root-nodule development (Lambais and Mehdy, 1993; Przysiecka et al., 2015). So, the opposite low expression of *OjCHI* in root suggests that OjCHI is not a leguminous CHI and probably responsible for the formation of anthocyanin and proanthocyanidin. Correspondingly, we did note *OjCHI* transcripts in different tissues were strongly consistent with the accumulation of anthocyanin, which further verified its function in anthocyanin biosynthesis (Figure 5C). Furthermore, transcript profiles of *OjCHI* gene during flower developmental stages were also performed (Figure 5B). At different stages of flower development, it was found that *OjCHI* showed higher expression in the earlier bud than the fully opened flowers which matched the accumulation pattern of anthocyanin (Figure 5D), consistently, this expression pattern was also observed in *Gentiana triflora* (Nakatsuka et al., 2005). Transcript accumulation of *CHIs* in flowers has already been reported in tulips, Chrysanthemum, as well as petunia, and the studies demonstrate that different expression patterns of *CHIs* are a determinant for petal color variations (Chen et al., 2012; Yuan et al., 2013; Akhar et al., 2016). For instance, high level expression of *CHI* gene conferred red petals in petunia, while inhibiting its expression made tobacco petal coloration turn to yellow (Nishihara et al., 2005; Akhar et al., 2016). Thus, these results suggest that transcript expression of *OjCHI* may regulate the accumulation of pigment during *O. japonica* flower development.

Functionality of *OjCHI* was further investigated through its over-expression in Arabidopsis *tt5* mutant. The results present in Figure 7 showed that *OjCHI* could recover the color phenotypes of seed, cotyledons and hypocotyls of *tt5* mutant and rescue the deficiency of flavonoid accumulation, which demonstrated the capacity of OjCHI in catalyzing the cyclization of endogenous chalcone to produce flavonols, proanthocyanidins and anthocyanins. Likewise, such *in vivo* activity of *CHI* gene from alfalfa and *Ipomoea batatas* also obtained the same results, suggesting that CHI proteins involved in flavonoid metabolism are functionally exchangeable among distantly related plants (Liu et al., 2002; Guo et al., 2015). Meanwhile, these findings unambiguously indicate the value of Arabidopsis mutants as a useful and convenient system for assaying the function of uncharacterized genes from other plants. But unexpectedly, one previous study reported that mutant maize CHI (having 3–5% activity compared to wild-type CHI) could also complement the phenotypes of *tt5* mutant, and this raise the possibility that CHI may have functions other than catalyzing naringenin chalcone, perhaps serving as transporters and/or chaperons during flavonoid biosynthesis, or functioning as a structural scaffold for enzymes in flavonoid pathway (Dong et al., 2001; Ralston et al., 2005).

## Conclusion

In conclusion, in this study we have functionally identified one *CHI* gene, *OjCHI*, which plays a significant role in anthocyanin biosynthesis in *O. japonica*. Integrative expression analysis indicated that *OjCHI* had tissue-specific expression and its transcription pattern coincided with the change of anthocyanin accumulation not only in different tissues but also in developing flowers. *In vitro* enzyme assays of recombinant OjCHI confirmed its predicted function in the biosynthesis of anthocyanin. Moreover, the *in vivo* genetic analysis of *OjCHI* in Arabidopsis *tt5* mutant further proved its role in proanthocyanidin and anthocyanin biosynthesis. Therefore, the findings from this article will advance our understanding of the molecular mechanisms of anthocyanin biosynthesis in *O. japonica*, and also provide a basis for flavonoid manipulation studies through using molecular approaches in the future.

## Data Availability

All datasets for this study are included in the manuscript and the Supplementary Files.

## Author Contributions

ZJ and YY conceived and designed the study. WS, HS, HX, and XT conducted the experiments, analyzed and interpreted the data, and wrote the manuscript. WS, MT, ZJ, and YY revised the manuscript critically. All authors read and approved the final manuscript.

## Conflict of Interest Statement

The authors declare that the research was conducted in the absence of any commercial or financial relationships that could be construed as a potential conflict of interest.

## Footnotes

1 https://sg.idtdna.com/primerquest/Home/Index
