# Supplementary material for: Chalcone Isomerase a Key Enzyme for Anthocyanin Biosynthesis in Ophiorrhiza japonica
Source: Front Plant Sci. 2019 Jul 9;10:865. doi: 10.3389/fpls.2019.00865 (PMC6629912; doi:10.3389/fpls.2019.00865)

***Supplementary Material***

**SUPPLEMENTARY TABLES AND FIGURES**

**Supplementary Table S1** **List of primers used for *OjCHI* isolation and characterization**

| **Primer** | **Sequence 5’-3’** |
| --- | --- |
|  |  |
| OjCHIF1 | AATGTCTGGATCATCAAT |
| OjCHIR1 | TCTTCAGGCATTTCTTTA |
| OjCHIF2 | CACGGGGGACTCTAGAATGTCTGGATCATCAATCTCCATC |
| OjCHIR2 | GACCACCCGGGGATCCCAGTTGCTGTCCCTCTTTGAAG |
| OjCHIF-RT | CAGTTAACGCTCCTGGTTCTAA |
| OjCHIR-RT | GCAGACTCTTCCAAGTACACTC |
| ActinF | AGGAGCTTGAGACAGCAAAG |
| ActinR | GGGCATCGGAATCTCTCATT |
| OjCHIF3 | CGGGATCCATGTCTGGATCATCAATC |
| OjCHIR3 | CGGAATTCTTACAGTTGCTGTCCCTC |
| OjCHIF4 | GC TCTAGAATGTCTGGATCATCAATC |
| OjCHIR4 | CG GGATCCTTACAGTTGCTGTCCCTC |
|  |  |

* Restriction enzyme site are underlined

**Supplementary Table S2 HPLC-DAD and HPLC-ESI-MS analysis of anthocyanin and flavonol extracts of the wild-type Arabidopsis and *OjCHI* over-expressing lines**

| Peak number | Identifacation/tentative  identification | Retention time (min) | λmax (nm) | ESI-MS (m/z) | References |
| --- | --- | --- | --- | --- | --- |
| 1 | Cyanidin 3-*O*-[2*''*-*O*-(xylosyl) 6*''*-*O*-(*p*-*O*-(glucosyl) *p*-coumaroyl) glucoside] 5-*O*-[6*''''*-*O*-(malonyl) glucoside] | 47.276 | 265  530 | 287.0  1137.2 | Takayuki Tohge, Yasutaka Nishiyama et al., 2005 |
| 2 | Cyanidin 3-*O*-[2*''*-*O*-(6*'''*-*O*-(sinapoyl) xylosyl) 6*''*-*O*-(p-*O*-(glucosyl)-*p*-coumaroyl) glucoside] 5-*O*-(6*''''*-*O*-malonyl) glucoside | 48.573 | 257  532 | 287.1  1343.1 | Stephen J. Bloora, Sharon Abrahamsb., 2002 |
| 3 | Pelargonidin derivatives | 53.851 | 277  525 | 271.2 |  |
| 4 | Cyanidin derivatives | 54.998 | 275  532 | 287.1 |  |
| 6 | Kaempferol 7-*O*-rhamnopyranoside | 23.038 | 258  351 | 287.0  433.1 | Jin-Ying Gou, Felipe F. Felippes et al., 2011 |
| 7 | Quercetin 3-*O*-rhamnoside 7-*O*-rhamnoside | 27.547 | 263  352 | 303.1  449.0 | Graham., 1998 |
| 8 | Kaempferol derivatives | 36.431 | 259  355 | 287.2 |  |
| 10 | Kaempferol 3-*O*-glucoside 7-*O*-rhamnoside. | 47.491 | 263  347 | 287.1  433.2 | Takayuki Tohge, Yasutaka Nishiyama et al., 2005 |
| 12 | Kaempferol 3-*O*-rhamnoside 7-*O*-rhamnoside | 54.285 | 267  349 | 286.9  433.1 | Jin-Ying Gou, Felipe F. Felippes et al., 2011 |

There was not obvious aglycone ion corresponded to peak 5, peak 9 and peak 11, thus these three peaks were not used for quantitative analysis.

Reference list for Table S2

1. Tohge T, Nishiyama Y, Hirai MY, Yano M, Nakajima J, Awazuhara M, Inoue E, Takahashi H, Goodenowe DB, Kitayama M, Noji M, Yamazaki M, Saito K (2005) Functional genomics by integrated analysis of metabolome and transcriptome of *Arabidopsis* plants over-expressing an MYB transcription factor. Plant J 42: 218-235.

2. Stephen J. Bloor^a^, Sharon Abrahams^b^ (2002) The structure of the major anthocyanin in Arabidopsis thaliana. Phytochemistry 59: 343-346.

3. Gou JY, Felippes FF, Liu CJ, Weigel D, Wang JW (2011) Negative regulation of anthocyanin biosynthesis in Arabidopsis by a miR156-targeted SPL transcription factor. Plant Cell 23: 1512-1522.

4. Graham TL (1998) Flavonoid and flavonol glycoside metabolism in *Arabidopsis*. Plant physiol Biochem 36: 135-144.

**Supplementary Figure S1. Anthocyanin and flavonol component analysis in different tissues and flowers at different developmental stages of *O. japonica*.** A. Different tissues. B. Flowers at different developmental stages. Data represent means ± SD of three biological replicates.


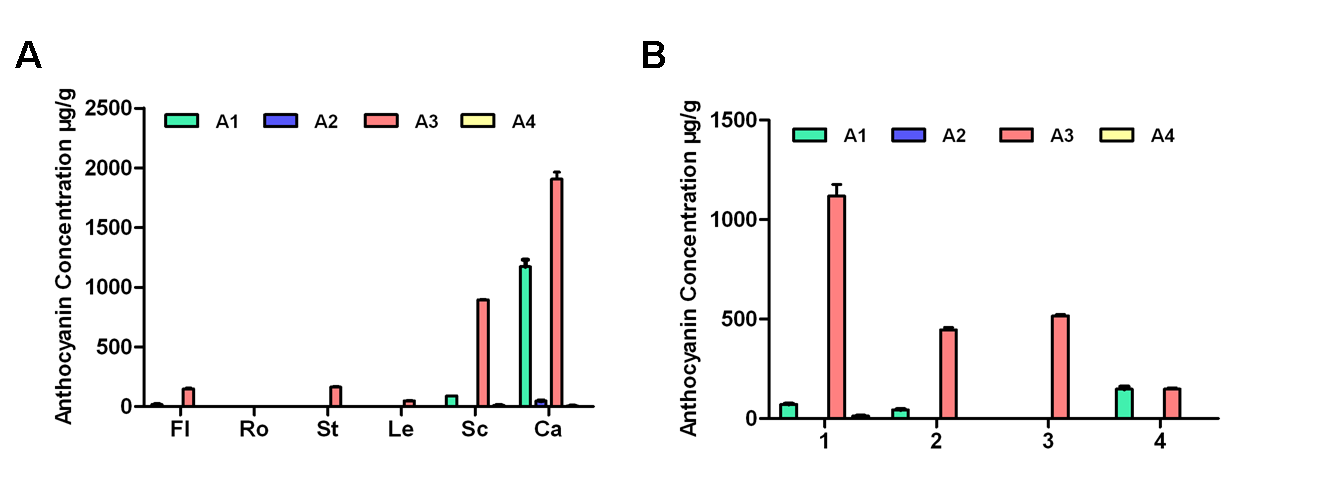

Supplement: Supplementary file 1 [file Data_Sheet_1.doc]
